# Association between platelet lymphocyte ratio and neutrophil lymphocyte ratio and clinical outcomes following carotid endarterectomy

**DOI:** 10.1590/1677-5449.202201222

**Published:** 2023-08-28

**Authors:** Vinicius Adorno Gonçalves, Martin Andreas Geiger, Danilo Augusto Sarti, Ana Terezinha Guillaumon

**Affiliations:** 1 Universidade Estadual de Campinas – UNICAMP, Faculdade de Ciências Médicas, Hospital de Clínicas, Campinas, SP, Brasil.; 2 National University of Ireland, Hamilton Institute, Maynooth, Ireland.

**Keywords:** platelet count, atherosclerosis, carotid stenosis, carotid endarterectomy, mortality, contagem de plaquetas, aterosclerose, estenose das carótidas, endarterectomia carotídea, mortalidade

## Abstract

**Background:**

Approximately 30% of stroke cases result from carotid disease. Although several risk factors for complications after carotid endarterectomy have been identified, the existence of a biomarker that can estimate postoperative risk in these patients has not yet been proven.

**Objectives:**

This study aimed to investigate correlations between the platelet-lymphocyte ratio (PLR) and the neutrophil-lymphocyte ratio (NLR) and postoperative clinical outcomes in patients undergoing carotid endarterectomy.

**Methods:**

A retrospective study was conducted, including 374 patients who underwent carotid endarterectomy between 2002 and 2019 due to moderate to high extracranial internal carotid artery stenosis. Their platelet-lymphocyte ratio and neutrophil-lymphocyte ratios were obtained from the same blood samples.

**Results:**

There was a statistically significant correlation between the PLR and the occurrence of restenosis (p < 0.01) and acute myocardial infarction (AMI) after endarterectomy (p = 0.03). Additionally, there was a statistically significant correlation between the PLR and the combined outcomes stroke and/or AMI and/or death (p = 0.03) and stroke and/or AMI and/or death and/or restenosis (p < 0.01). However, there were no significant correlations between NLR and these outcomes (p = 0.05, p = 0.16).

**Conclusions:**

The platelet-lymphocyte ratio proved to be a useful test for predicting occurrence of strokes, acute myocardial infarctions, and deaths during the postoperative period after carotid endarterectomy. It was also associated with the risk of postoperative restenosis.

## Resumo

**Contexto:**

Aproximadamente 30% dos casos de acidente vascular cerebral (AVC) resultam de doença carotídea. Embora vários fatores de risco para complicações pós-endarterectomia carotídea tenham sido identificados, ainda não foi comprovada a existência de um biomarcador que possa estimar o risco pós-operatório nesses pacientes.

**Objetivos:**

Correlacionar o índice plaqueta-linfócito (IPL) e o índice neutrófilo-linfócito (INL) com os desfechos clínicos pós-operatórios em pacientes submetidos a endarterectomia carotídea.

**Métodos:**

Estudo retrospectivo que incluiu 374 pacientes submetidos a endarterectomia carotídea, entre 2009 e 2019, por estenose extracraniana da artéria carótida interna. O IPL e o INL foram calculados, tendo sido obtidos das mesmas amostras de sangue.

**Resultados:**

Houve correlação estatisticamente significativa entre IPL e presença de reestenose (p<0,01) e infarto agudo do miocárdio (IAM) após endarterectomia (p=0,03). Os desfechos combinados AVC e/ou IAM e/ou óbito e AVC e/ou IAM e/ou óbito e/ou reestenose apresentaram, respectivamente, correlação estatisticamente significativa com o IPL (p=0,03; p<0,01) e não significativa com o INL (p=0,05; p=0,16).

**Conclusões:**

O IPL mostrou-se um teste útil, capaz de predizer os desfechos de AVC e/ou IAM e/ou óbito em pacientes no pós-operatório de endarterectomia carotídea, relacionando-se também com risco de reestenose pós-operatória.

## INTRODUCTION

Stroke is the third leading cause of death and dysfunction worldwide. Approximately 30% of cases result from carotid stenosis.^1-5^ Similar to coronary heart disease, inflammatory activity plays a significant role in the development and progression of atherosclerotic carotid disease.^3^

Blood counts can provide valuable information in patients with carotid disease. White cell counts and their subtypes serve as systemic markers of inflammatory activity.^6^ Previous studies have demonstrated that elevated levels of neutrophils and platelets together, along with reduced levels of lymphocytes, are associated with vascular diseases.^2,3,7,8^ The neutrophil-lymphocyte ratio (NLR) and platelet-lymphocyte ratio (PLR) have been proposed as markers that combine information on the atherosclerotic processes (hemostasis and inflammation) that can lead to plaque rupture.^7,8^

Although several risk factors for complications after carotid endarterectomy have been identified in the literature, the existence of a biomarker that can estimate postoperative risk in these patients has not yet been proven. Such a biomarker would be particularly useful when determining the optimal treatment approach for high-risk patients or asymptomatic patients without prior ischemic events, aiding in the process of deciding between surgery and medical therapy. Use of indices based on white cell count data, such as NLR and PLR, constitutes a simple, widely applicable, and cost-effective method for determining markers of pro-inflammatory activity.^6^

## MATERIALS AND METHODS

### Data source

This retrospective study enrolled 374 patients who underwent carotid endarterectomies because of moderate to high (>70%) extracranial internal carotid artery stenosis, as determined on computed tomography. All surgeries, postoperative care, and outpatient follow-up were performed by the same vascular surgeons, employing conventional techniques with primary closure, patching, semi-eversion, or eversion, based on surgeon preference. The study included a continuous, consecutive series of single-center cases.

Clinical and demographic data were collected from hospital medical records, and laboratory data from venous blood sampling, including hematologic and biochemical parameters, were recorded. The PLR and NLR were calculated as the ratio of platelets to lymphocytes and the ratio of neutrophils to lymphocytes and were obtained from the same blood sample (measurements in units per cubic millimeter). The most recent blood count conducted within a period not exceeding 6 months prior to surgery was selected for analysis.

Patients with elevated white cell blood counts were excluded to minimize other confounding factors, such as infection, hematological diseases, and other illnesses. Postoperative PLR and NLR were not analyzed because white cell blood counts are often elevated in response to the surgery. If patients had multiple laboratory results before surgery, the sample drawn immediately preceding surgery was used. All patients had these laboratory tests as part of their routine preoperative testing. The project adhered to the Strengthening the Reporting of Observational Studies in Epidemiology (STROBE) checklist for cohort studies and all 15 STROBE items were analyzed and validated.

### Study population

**Figure 1:**
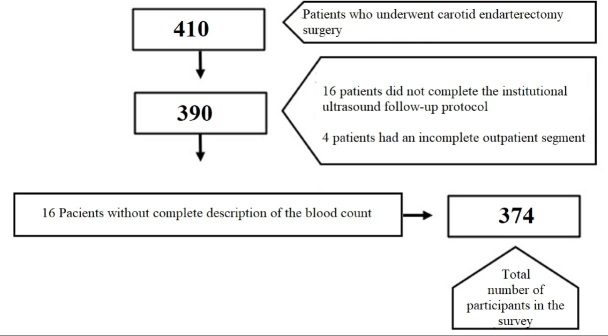
Flowchart of patients included in and excluded from the study.

A total of 410 patients who underwent carotid endarterectomy surgery between 2002 and 2019 were initially selected. The institutional protocol required follow-up at the vascular surgery carotid outpatient clinic, where patients underwent control ultrasound at regular intervals postoperatively (1, 3, 6, 9, 12, 18, and 24 months), performed by the same medical team. Restenosis was defined as presence of >70% stenosis identified in a postoperative imaging exam starting 1 month after surgery. Symptomatic patients (with ischemic events within the last 6 months) with >70% carotid stenosis, asymptomatic patients with >80% carotid stenosis, and patients with preoperative imaging exams were included. Patients with inconclusive preoperative imaging exams, incomplete study segments, incomplete blood count descriptions, or incomplete data were excluded. A total of 36 patients (8.78%) were excluded, as illustrated in Figure 1.

### Endpoints

Primary: To examine the correlation between the platelet-lymphocyte ratio (PLR) and the neutrophil-lymphocyte ratio (NLR) and postoperative clinical outcomes in patients undergoing carotid endarterectomy. The clinical outcomes of interest include restenosis, bleeding, sepsis, stroke/transient ischemic attack, cranial nerve injury, acute myocardial infarction, and death identified during the study follow-up period.

Secondary: To determine the clinical and epidemiological profile of patients undergoing carotid endarterectomy.

### Statistical analysis

An exploratory analysis was initially conducted to ensure the consistency of the database. Descriptive statistics such as means and medians were calculated. Logistic regression models were then fitted to assess the impact of NLR and PLR on the outcomes of interest. Each outcome was considered as a binary response variable in a separate logistic regression model. Both PLR and NLR were included as explanatory variables in all the models, with a significance level of p < 0.05. Logistic regression was chosen over survival analysis models like Cox regression because the focus was on the odds ratios of the outcomes given NLR and PLR, rather than the long-term effect of time. Odds ratios were calculated after fitting the models and the following categories were considered: <1, indicating a reduction in the risk of the outcome, = 1, indicating no relationship between the predictor and the outcome, and >1, indicating an increase in the risk of the outcome.^9^ The statistical analysis was conducted using R version 4.0. The minimum sample size was calculated using G*power version 3.1, considering a non-parametric sample for repeated measures with 2 groups and 2 times, a significance level of 5%, a power of 95%, and an effect size of 50%.

### Ethics committee approval

The protocol was approved by the Ethics Committee (opinion number: 4.164.347, CAAE: 30630220.7.0000.5404). The ethics committee waived the requirement for informed consent.

## RESULTS

### Epidemiology

A majority of the total sample of 374 patients were male (70.3%) and mean age was 69.04 years. Most patients had a history of cerebrovascular events (72.7%) and the majority of surgeries were performed on asymptomatic patients (62.3%). Table 1 shows the patients’ clinical and epidemiological profile and the prevalence of comorbidities. The mean follow-up time was 43.5 months (CI 95% 42.3-44.6) and 296 patients (79.1%) completed the 48-month follow-up. All patients underwent preoperative cardiac assessments and additional tests as required by the institutional protocol. According to risk classification, 13.6% were considered low risk, 68.2% moderate risk, and 18.2% high risk for cardiovascular events.

**Table 1:** Sample description and profile of comorbidities identified in the preoperative period in patients undergoing carotid endarterectomy surgery.

	**Patients (n=374)**	**Interquartile range**
Age, years (mean)	69.04	64 - 75
Male (%)	263 (70.3)	-
Systemic Arterial Hypertension (%)	335 (89.6)	-
Diabetes Mellitus (%)	151 (40.4)	-
Dyslipidemia (%)	190 (50.8)	-
Hypothyroidism (%)	39 (10.4)	-
Smoking (%)	275 (73.5)	-
COPD (%)	46 (12.3)	-
Alcoholism (%)	49 (13.1)	-
Previous AMI* (%)	61 (16.3)	-
Heart failure (%)	33 (8.8)	-
CKD (%)	47 (12.6)	-
LEAD (%)	123 (32.9)	-
Cerebrovascular Event (%)	272 (72.7)	-
Stroke (%)	212 (56.7)	-
TIA (%)	84 (22.5)	-
Stenosis, mean (%)	77.7	70 - 92
Previous Contralateral Surgery (%)	43 (11.5)	-
Contralateral Occlusion (%)	19 (5.1)	-
Symptomatic (%)	141 (37.7)	-
Asymptomatic (%)	233 (62.3)	-

COPD = chronic obstructive pulmonary disease; AMI = acute myocardial infarction; CKD = chronic kidney disease; LEAD = lower extremity arterial disease; TIA = transient ischemic attack.

Symptomatic: patients with cerebrovascular ischemic events in the past 6 months.

Stenosis was graded based on the preoperative tomography exam.

### Postoperative results

Regarding the laterality of the approach, 46.8% of the endarterectomies were performed on the left internal carotid artery, while 53.2% were performed on the right. The classic, semi-eversion, and eversion techniques were used in 27.3%, 70%, and 2.7% of the cases, respectively. A shunt was used in 1.9% of patients, and a bovine pericardial patch was used for closure in 4.8% of patients. General anesthesia was chosen for 67.9% of the cases, while locoregional anesthesia was chosen for 32.1%

In the main analysis of postoperative outcomes, 9.1% of patients presented restenosis during follow-up (restenosis was defined as the presence of stenosis identified in a postoperative imaging exam with onset 1 month after surgery), 3.5% had AMI (Acute myocardial infarction), 6.9% experienced stroke or TIA (Transient ischemic attack), and 3.5% died. The combined rate of stroke and/or AMI and/or death was 12%, and the combined rate of stroke and/or AMI and/or death and/or restenosis was 19.2%. These outcomes are listed in Table 2.

**Table 2:** Postoperative outcomes of patients undergoing carotid endarterectomy surgery.

	**Patients (n=374)**
Restenosis* (%)	34 (9.1)
Average time to restenosis (months)	10.4 (mean)/ 8.5 (median)
Reintervention for restenosis (%)	6 (1.6)
Bleeding/bruising (%)	28 (7.5)
Reintervention for bruising (%)	20 (5.3)
SEPSIS (%)	8 (2.1)
Stroke/TIA (%)	26 (6.9)
Cranial nerve injury (%)	11 (2.9)
AMI** (%)	13 (3.5)
Death (%)	13 (3.5)
Combined outcomes	
Stroke and/or AMI and/or Death (%)	45 (12)
Stroke and/or AMI and/or Death and/or Restenosis (%)	72 (19.2)

* Restenosis defined as >70% stenosis identified during postoperative follow-up.

AMI = acute myocardial infarction; TIA = transient ischemic attack; SEPSIS = *life-threatening organ dysfunction caused by a dysregulated host response to infection.*

In the multivariate analysis, we separately modeled preoperative PLR and NLR to determine whether their associations with outcomes persisted through the perioperative period. Table 3 demonstrates five separate multivariate models identified during study follow-up (restenosis, sepsis, cerebrovascular accident, acute myocardial infarction, and death), each adjusted for clinically relevant variables (age, sex, disease severity, and pre-existing conditions). Sepsis was defined as a suspected or documented infection accompanied by organ dysfunction.

**Table 3:** Association between PLR and NLR and postoperative outcomes and complications in patients undergoing carotid endarterectomy surgery. Each response variable was studied with univariate analysis considering PLR and NLR as explanatory variables.

**Variable**	**Explanatory Variable**	**Odds Ratio (IC 95% +/-)**	**p. value**
Restenosis*	PLR	1.02 (0.02-2.02)	<0.01
Restenosis*	NLR	0.87 (0-2.04)	0.41
Reintervention for restenosis	PLR	0.99 (0-2.01)	0.48
Reintervention for restenosis	NLR	0.63 (0-2.43)	0.46
SEPSIS	PLR	1.01 (0.01-2.01)	0.01
SEPSIS	NLR	1.08 (0-2.33)	0.73
Stroke	PLR	1.01 (0-2.01)	0.05
Stroke	NLR	1.33 (0.17-2.49)	0.06
AMI*	PLR	1.01 (0-2.01)	0.19
AMI*	NLR	1.14 (0-2.37)	0.54
Death	PLR	1.01 (0-2.02)	0.06
Death	NLR	1.16 (0-2.38)	0.44
AMI and/or stroke and/or death	PLR	1.01 (0-2.01)	0.03
AMI and/or stroke and/or death	NLR	1.31 (0-2.46)	0.05
AMI and/or stroke and/or death and/or restenosis	PLR	1.01 (0-2.01)	<0.01
AMI and/or stroke and/or death and/or restenosis	NLR	1.21 (0-2.34)	0.16

* Restenosis defined as >70% stenosis identified during postoperative follow-up.

AMI = acute myocardial infarction; SEPSIS = *life-threatening organ dysfunction caused by a dysregulated host response to infection.*

Preoperative PLR was found to be associated with restenosis (p<0.01), sepsis (p=0.01), the combined outcomes of AMI and/or stroke and/or death (p=0.03), and the combined outcomes of AMI and/or stroke and/or death and/or restenosis (p<0.01). Other covariates in the model that were associated with the outcomes under study are detailed in Table 3.

## DISCUSSION

NLR and PLR are involved in different immunological pathways and activate the nonspecific inflammatory response by increasing the neutrophil or platelet count.^2,10,11^ PLR and NLR are markers that combine information on hemostasis and inflammation, with high sensitivity for demonstrating the inflammatory process.^3,7,12-16^ Several studies suggest that NLR and PLR can be markers of inflammatory activity in various diseases.^12,14-18^ Perioperative NLR and PLR levels are significantly correlated with patient morbidity and mortality rates in those undergoing percutaneous surgery, cardiac surgery, and vascular surgery procedures.^17^

Platelets promote neovascularization, releasing thromboxanes, pro-inflammatory chemokines, growth factors such as transforming growth factor b1, endothelial growth factor, and platelet-derived growth factor, and cytokines, all of which participate in the vascular inflammatory process and thrombosis. They induce release of cytokines and interact with different types of immune system cells, including neutrophils, T lymphocytes, NK lymphocytes, and macrophages. Activated platelets participate in thrombus formation in response to atherosclerotic plaque rupture or endothelial cell erosion, promoting the development of atherothrombotic disease or adverse cardiovascular events.^2,7,8,11,17^

Neutrophils represent the largest subclass of leukocytes. They promote cell proliferation and angiogenesis and produce vascular endothelial growth factor (VEGF). On the other hand, the lymphocyte count is an indicator of physiological stress and is inversely associated with inflammation. A low lymphocyte count, which indicates suppression of the immune and inflammatory process, increases cardiovascular risk and mortality.^7,8^

Lymphocytes produce cytokines that inhibit cell proliferation and promote cell death. Lymphocyte apoptosis has been observed in atherosclerotic lesions involved in atherosclerotic plaque growth, lipid core development, plaque rupture, and thrombosis.

Carotid artery stenosis is a peripheral vascular disease that can cause severe neurological consequences when symptomatic.^8,9,19^

To be classified as symptomatic, it must be related to ischemic events in the last 6 months. Stroke can be prevented by surgical excision of the atherosclerotic plaque with carotid endarterectomy or stent angioplasty. The benefit of carotid revascularization for a patient depends on the balance between the long-term risk of vascular complications on medical treatment and the risk of periprocedural complications.^19^ Indications for carotid endarterectomy in symptomatic patients began to be studied in the 1990s, demonstrating good results for patients with severe stenoses compared to clinical treatment.^2,4^ For medical indications, in addition to the patient’s symptoms and comorbidities, the severity of the stenosis and the anatomical and psychological characteristics of the plaque are also taken into account. Surgery is mainly recommended in symptomatic cases with 70% or more stenosis or in asymptomatic cases with 60% or more stenosis combined with other risk factors for plaque embolization.^4,5^ The maximum benefit in preventing a future cerebrovascular ischemic event has been shown in the first 2 weeks after the initial ischemic event.^1,5^

Endarterectomy is an effective long-term stroke prevention strategy in symptomatic patients. However, controversies still remain in asymptomatic patients compared to clinical treatment, with important differences between guidelines around the world.^3,18^ In our study, 37.7% of patients were considered symptomatic. There was a statistically significant correlation between this variable and the IPL (p<0.01), demonstrating that these patients with recent cerebrovascular ischemic events were in a more exacerbated inflammatory state, raising the hypothesis that asymptomatic patients with the same biological profile may be at a greater risk of developing ischemic events. This could serve as a marker for medical indication and prioritization of patients on waiting lists.

The mean age of the patients in our study was 69 years and they often had multiple comorbidities, as detailed in Table 1. The prevalence of previous cardiovascular and cerebrovascular events was also high, with 16.3% of patients reporting previous AMI and 72.7% reporting previous cerebrovascular events, such as stroke (56.7%) and TIA (22.5%).

The postoperative complications related to carotid endarterectomy on which the literature contains the most significant evidence include stroke, AMI, carotid restenosis, and death.^19-23^ Carotid endarterectomy also involves local risks: cranial nerve injury and postoperative cervical hematoma are well-recognized potential complications.^24^ Infection with progression to sepsis is a particularly dangerous postoperative complication and is essentially related to increased morbidity and mortality in these patients. In our study, 2.1% of endarterectomy patients progressed to sepsis in the postoperative period (the majority with a pulmonary focus), and this variable had a statistically significant correlation with the PLR (p=0.01).

Postoperative stroke is defined as development of a new focal neurologic deficit or worsening of an existing deficit after carotid endarterectomy. Microembolism and macroembolism are the main causes of cerebral ischemia in the perioperative period after carotid endarterectomy.^21^ Patients with perioperative stroke have an almost 40-fold higher risk of mortality within 30 days.^21,22^ Some studies have reported that uncontrolled systolic blood pressure is associated with perioperative stroke.^23,24^

In our study, the rate of stroke was 6.9%, the majority of which (53.8%) occurred more than 24 hours after the procedure. However, there were no statistically significant correlations between the NLR or PLR and the presence of postoperative stroke or TIA, with p values of 0.05 and 0.06, respectively.

Restenosis can develop due to neointimal hyperplasia or recurrent atherosclerosis after surgery. The literature reports a restenosis rate of 6.3% within 2 years’ follow-up after carotid endarterectomy.^21^ In our study, with a 4-year follow-up period, the rate of significant postoperative carotid restenosis (>70%) was 9.1% and mean restenosis time was 10.4 months. Six patients (1.6%) required reintervention for restenosis, with carotid angioplasty with stenting. We found a statistically significant correlation between the PLR and occurrence of restenosis (p<0.01), suggesting an association with the chronic inflammatory process. However, there were no correlations between the NLR or the PLR and the need for reintervention for restenosis.

Approximately 28% of patients undergoing carotid endarterectomy have severe coronary artery disease, which puts them at risk of postoperative acute myocardial infarction (AMI).^21^ In our study, the rate of AMI after endarterectomy was 3.5%, showing a statistically significant correlation with the PLR (p=0.03). Calculating the PLR can be useful for identifying patients at higher risk of cardiovascular events. The postoperative mortality rate was 3.5% for our patients, and there were no statistical correlations between the PLR or the NLR and mortality.

In our study, 86.4% of the patients were classified as having a moderate or high risk of postoperative cardiovascular events, which could explain the high rates of AMI and death. Perioperative cardiovascular complications are important causes of morbidity and mortality associated with non-cardiac surgery. The risks are linked to both patient-related and procedure-related factors, such as clinical and comorbid conditions and surgery-specific characteristics. Assessments and strategies to improve clinical outcomes are increasingly sought.^25,26^

Surgical procedures themselves carry inherent risks beyond patient comorbidities for various reasons, including blood loss, fluid dynamics, inflammation, patient positioning, ventilation/perfusion imbalances, and other acute physiological changes.^26^ These findings can be attributed to the high prevalence of comorbidities in patients with carotid artery disease, especially those referred to high complexity services like ours.

Systemic inflammation has been implicated in the development of cognitive dysfunction following carotid endarterectomy. However, contrary to our study findings, other studies suggest that the NLR is a readily available marker of systemic inflammation and that patients with elevated preoperative NLR have an increased risk of cognitive dysfunction after carotid endarterectomy (CEA).^27^

Significant improvements in medical therapy have reduced the incidence of development of symptoms from carotid stenosis to less than 1%, making best medical therapy (BMT) an effective and safe treatment for high-risk patients with carotid artery disease.^28^ Although we did not find any relationship between the outcomes and the NLR, other literature suggests that the risk-benefit ratio of CEA in asymptomatic patients with a high NLR may favor BMT, since CEA is best suited for low-risk patients in good health. An NLR >3.0 may be associated with an increased risk of late stroke or death after CEA for asymptomatic carotid artery stenosis.^28^

The combined outcome of stroke, AMI, and/or death was 12%, showing statistically significant association with the PLR (p=0.03) and borderline significance with relation to the NLR (p=0.05). The rate of the combined outcome of stroke, AMI, and/or death, and/or restenosis was 19.2%, also demonstrating a statistical correlation with the PLR (p<0.01), but no significant correlation with the NLR (p=0.16).

This study has several limitations that must be acknowledged. It is a retrospective, single-center, registry study, relying on information collected solely from medical records without randomization. Due to service flow constraints, it was not possible to compare the surgical group with a control group on BMT. Cut-off values for NLR and PLR could not be determined statistically and the literature lacks consensus on the appropriate cut-off values for these indices. Further studies with larger numbers of patients are needed to confirm our findings and establish cut-off points for the NLR and PLR, bringing use of these indices in clinical practice closer. Additionally, there are no validated inflammatory markers in the literature, which prevented us from validating the scores by comparing them with others for evaluating clinical outcomes after carotid endarterectomy.

## CONCLUSION

In this retrospective study analyzing markers associated with patients treated for carotid artery disease, the PLR emerged as a valuable test for predicting outcomes of stroke, AMI, and/or death in the postoperative period after carotid endarterectomy. The PLR was also found to be associated with the risk of postoperative restenosis. However, the NLR did not demonstrate statistical significance for predicting these events in our analysis.
